# Combined aerobic and resistance exercise training attenuates cardiac dysfunctions in a model of diabetes and menopause

**DOI:** 10.1371/journal.pone.0202731

**Published:** 2018-09-07

**Authors:** Iris Callado Sanches, Morgana Buzin, Filipe Fernandes Conti, Danielle da Silva Dias, Camila Paixão dos Santos, Raquel Sirvente, Vera Maria Cury Salemi, Susana Llesuy, Maria-Cláudia Irigoyen, Kátia De Angelis

**Affiliations:** 1 Human Movement Laboratory, Sao Judas Tadeu University (USJT), São Paulo, Brazil; 2 Laboratory of Translational Physiology, Universidade Nove de Julho (UNINOVE), São Paulo, Brazil; 3 Hypertension Unit, Heart Institute (InCor), School of Medicine, University of São Paulo, São Paulo, Brazil; 4 Facultad de Farmacia y Bioquimica, Universidad de Buenos Aires, Buenos Aires, Argentina; 5 Departament of Physiology, Federal University of Sao Paulo (UNIFESP), São Paulo, Brazil; Universita degli Studi Magna Graecia di Catanzaro Scuola di Medicina e Chirurgia, ITALY

## Abstract

The study aimed at evaluating the effects of combined aerobic and resistance exercise training on cardiac morphometry and function, oxidative stress and inflammatory parameters in diabetic ovariectomized rats. For this, female Wistar rats (10 weeks-old) were divided into 4 groups (n = 8): euglycemic (E), diabetic (streptozotocin, 50 mg/kg, iv) (D), diabetic ovariectomized (DO) and trained diabetic ovariectomized (TDO). The combined exercise training was performed on a treadmill and in a ladder adapted to rats (8 weeks, at 40–60% of maximal capacity). The left ventricle (LV) morphometry and function were evaluated by echocardiography. Oxidative stress and inflammatory markers were measured on ventricles tissue. The sedentary diabetic animals (D and DO) showed impaired systolic and diastolic functions, as well as increased cardiac overload, evaluated by myocardial performance index (MPI- D: 0.32 ± 0.05; DO: 0.39 ± 0.13 vs. E: 0.25 ± 0.07), in relation to E group. Systolic and MPI dysfunctions were exacerbated in DO when compared to D group. The DO group presented higher protein oxidation and TNF-α/IL-10 ratio than D groups. Glutathione redox ratio (GSH/GSSG) and IL-10 were decreased in both D and DO groups when compared to E group. Exercise training improved exercise capacity, systolic and diastolic functions and MPI (0.18±0.11). The TDO group showed reduced protein oxidation and TNF-α/IL-10 ratio and increased GSH/GSSG and IL-10 in relation to the DO group. These results showed that combined exercise training was able to attenuate the cardiac dysfunctions, probably by reducing inflammation and oxidative stress in an experimental model of diabetes and menopause.

## Introduction

Estrogen deficiency, insulin resistance, hypertension, pro-inflammatory state and a sedentary lifestyle have been associated with the increased prevalence of cardiovascular disease in menopausal women [1]. In fact, heart disease is a major cause of mortality in women after menopause [2–7]. Moreover, the risk of diabetes mellitus (DM) increases after menopause [7], and DM increases cardiovascular risk in both pre- and postmenopausal women [6,8,9]. Indeed, cardiovascular events are the main cause of death among patients with type 2 diabetes [10]. Diabetes cardiomyopathy was described as chronic heart failure in diabetic patients and has been diagnosed as left ventricular (LV) failure in the absence of atherosclerosis and hypertension [11]. Ventricular failure is associated with calcium irregularities, apoptosis, fibrosis, and oxidative stress [12,13]. It should also be stressed that oxidative stress in the myocardium plays a key role in the pathogenesis of diabetic cardiomyopathy [14]. Both diabetic patients and animal models of diabetes mellitus display signs of oxidative damage in the heart tissue, such as lipid peroxidation, protein nitrosylation, and altered endogenous antioxidant enzyme levels [15].

Experimental models of diabetes, especially the streptozotocin (STZ) rat, have been used to help define the pathophysiology of this disorder [16]. Concerning menopause, the experimental model of bilateral surgical removal of ovaries (ovariectomy) has been used to mimic ovarian hormone deprivation [17–21]. However, both the role of the association of hyperglycemia and ovarian hormone deprivation in cardiac dysfunction and the role of oxidative stress and inflammation in this process remain unclear.

Since there is no cure for diabetes mellitus, finding effective therapeutic tools either to prevent the development of or treat diabetic cardiomyopathy is urgently needed to reduce the morbidity and mortality of the increasing diabetic population [22]. Additionally, over the past years, different approaches have been found to improve quality of life and cardiovascular health in menopausal women. In this sense, there is evidence that regular physical activity up-regulates the anti-oxidative defense system, attenuates the age-related increase in concentration of cellular reactive species of oxygen, and confers protection against oxidative stress-associated diseases [18,19]. Previous studies conducted by our group have shown that dynamic aerobic exercise training promoted body weight loss, resting bradycardia, normalization of arterial pressure, and improvement in baroreflex associated with decreased oxidative stress in normal and diabetic ovariectomized female rats [17,18]. Recently, we have demonstrated that both aerobic and resistance exercise training, either in combination or alone, promote an attenuation of cardiac morphometric dysfunction associated with reduced oxidative stress in an experimental model of diabetes and menopause. However, only dynamic aerobic exercise training was able to attenuate systolic and diastolic dysfunctions under this condition [19].

In exercise interventions discussed in the literature, however, resistance training appears to be the most efficient modality in the attenuation of sarcopenia, osteopenia, hepatic steatosis, and body composition changes promoted by ovarian hormone deprivation [23]. Furthermore, an important meta-analysis has found that resistance training plays a critical role in the control of risk factors, such as glycated hemoglobin and systolic arterial pressure, and should be indicated in the management of the diabetes [24].

In this sense, several guidelines suggest that the combination of these two approaches (aerobic plus resistance training) can maximize not only musculoskeletal benefits, but also cardiovascular risk in this population [24]. However, few studies have dealt with the cardiovascular effects of combined exercise training (aerobic + resistance) on cardiac dysfunction associated with diabetes and ovarian hormone deprivation. Therefore, the present study aims to test the hypothesis that aerobic training associated with resistance exercise training (in alternate days) can promote cardiac benefits associated with reduced inflammation and oxidative stress in STZ-induced diabetic ovariectomized rats.

## Methods

Experiments were performed using 32 female Wistar rats (10 weeks-old) obtained from Animal Facility of University of Sao Paulo, Brazil. The rats received standard laboratory chow (Nuvital, Colombo, Brazil) and water *ad libitum*. The animals were housed in individual cages in a temperature-controlled room (22°C) with 12-h dark-light cycle. Four experimental groups were used in this study: euglycemic *sham* operated (E, n = 8), diabetic (D, n = 8), diabetic ovariectomized (DO, n = 8) or undergoing combined exercise training protocol (TDO, n = 8). Considering the principles of 3Rs some data from E and DO animals were previously published (19). All rats were similarly treated with regard to daily manipulation. All surgical procedures and protocols were carried out in accordance with ARRIVE guidelines [25], and were approved by Universidade Nove de Julho Ethics Committee (protocol number AN001/08).

### Ovariectomy

10-week old animals from DO and TDO groups were anesthetized (80 mg/kg ketamine and 12 mg/kg xylazine, ip.), the oviduct was sectioned and the ovaries were removed as described in detail elsewhere [17,18,20]. At the same age, rats from E and D groups underwent a *sham* surgery. Immediately after surgery, the animals received antibiotic (benzetacil, 40.000U/Kg) and analgesic (tramadol chlorhydrate, 5mg/kg), and were supervised every 4 hours on the first day, and every 8 hours on the subsequent 3 days.

Data from our laboratory have demonstrated that the estrogen concentration, measured by immunoassay, was 39±7pg/ml in healthy female rats. However, in the present study estrogen concentration was non-detectable in ovariectomized studied groups (TKE21, Coat-A-Count Estradiol, Siemens Medical Solutions Diagnostics), thus confirming ovarian hormone deprivation [21].

### Diabetes

Five days after ovariectomy, the animals of the D, DO and TDO groups were made diabetic by a single injection of STZ (50 mg/kg IV; Sigma Chemical Co) dissolved in citrate buffer (pH 4.5) after 6 hours of fasting [20,21].

### Glycemia

Drops of blood (50 μL) were collected from the tip of the tail to measure glycemia 72 hours after STZ injection and at the end of the protocol with a Glucotest (Advantage, Roche Laboratories) [20,21].

### Combined exercise training

The combined exercise training consisted of aerobic and resistance training sessions on alternate days, carried out always in the same time of day (late afternoon), 5 days per week, during 8 weeks.

For the training prescription, maximum tests were performed on the treadmill (aerobic) and ladder adapted for rats (resistance), 5 days after the animal familiarization with the equipment, as described in detail in a previous publication [17,19,20]. These tests were performed three times: 1) at the beginning of the experiment, 2) in the fourth week, and 3) in the eighth week of the training protocol. The purpose was to determine the maximal physical capacity and exercise training intensity.

The aerobic exercise training was performed on a treadmill at low-moderate intensity (40–60% of the maximum speed obtained in the maximum running test), with duration of 60 minutes per session, without treadmill inclination.

The resistance training was carried out in a ladder adapted for animals, with a load attached to the tail base of the animal corresponding to the low-moderate intensity (40–60% of the load obtained in the maximum load test), with 15 climbs per session, a 1-minute time interval between climbs and duration of approximately 40 minutes per session.

Electric stimulation or food deprivation were not used to stimulate the rats run or climb during training sections. Only a touch of the hand at the base of the animal tail was used as a stimulus when necessary.

### Echocardiographic measurements

At the end of the protocol, echocardiography was performed by a double-blinded observer, under the guidelines of the American Society of Echocardiography. Rats were anesthetized (80 mg/kg Ketamine and 12 mg/kg Xylazine, ip.), and images were obtained with a 10–14 mHz linear transducer in a SEQUOIA 512 (ACUSON Corporation, Mountain View, CA).

The morphometric parameters analyzed were: left ventricular (LV) mass (adjusted for body weight), LV cavity in diastole (LVDIA) and relative wall thickness (RWT). The systolic function parameters were: velocity of circumferential fiber shortening (VCF) and fractional shortening (FS). Whereas the parameters of diastolic function were: LV isovolumetric relaxation time (IVRT) and peak E/peak A ratio (E/A). In addition, the myocardial performance index (MPI) was used as a global index of function, representing the myocardial effort to meet the demands of tissue perfusion, as described in detail elsewhere [19,26,27].

### Inflammatory mediators on cardiac tissue

One day after cardiac evaluations, the animals were killed by decapitation and the heart (ventricles) was immediately removed, rinsed in saline, and trimmed to remove fat tissue and visible connective tissue. IL-10 and TNF-α levels were determined using a commercially available ELISA kit (R&D Systems Inc.), in accordance with the manufacturer's instructions. ELISA was performed in 96-well polystyrene microplates with a specific monoclonal antibody coating. The threshold of sensitivity for the TNF-α and IL-10 assays was 15.0 pg/mL. Absorbance was measured at 540 nm in a microplate reader.

### Oxidative stress evaluations in cardiac tissue

The cardiac tissue (~0.5 g of ventricles) was cut into small pieces, placed in ice-cold buffer, and homogenized in an ultra-Turrax blender with 1 g tissue per 5 mL 150 mM KCl and 20 nM sodium phosphate buffer, pH 7.4. The homogenate was centrifuged at 600 g for 10 min at -26C. Protein was determined by the method of Lowry et al. [28], using bovine serum albumin as the standard.

Protein oxidation was measured by using a reaction of protein carbonyl groups with 2,4- dinitrofenylhydrazyne (DNPH) to form a 2,4-dinitrophenylhydrazone, which can be measured spectrophotometrically as previously described by Reznick & Packer [29]. The product of the reaction was measured at 360nm.

The quantification of superoxide dismutase (SOD) activity, expressed as U/mg protein, was based on the inhibition of the reaction between O2˙− and pyrogallol (34). Catalase (CAT) activity was determined by measuring the decrease in H_2_O_2_ absorbance at 240nm [30]. Glutatione peroxidase (GPx) activity was based on the consumption of NADPH at 480nm [31].

To determine GSSG and total glutathione concentration, tissue was homogenized in 2M perchloric acid and centrifuged at 1000g for 10min, and 2M KOH was added to the supernatant. The reaction medium contained 100mM phosphate buffer (pH 7.2), 2mM NADPH, 0.2U/mL glutathione reductase and 70μM 5,5’-dithiobis (2-nitrobenzoic acid). To determine the GSSG concentration, the supernatant was neutralized with 2M KOH and inhibited by the addition of 5μM N-ethylmaleimide. Absorbance was read at 420nm [32]. GSH values were determined from the total and GSSG concentration [33].

### Statistical analysis

Data are reported as means ± SEM. Levene’s test was used to assess variance homogeneity. Repeated measurement of ANOVA (body weight and glycemia) and One-way ANOVA (exercise capacity, cardiac morphometry and function, inflammation and oxidative stress) followed by the Student-Newman-Keuls *post-hoc* test were properly used for data analysis. Differences were considered significant at p≤0.05 for all tests.

## Results

### Metabolic parameters

Metabolic parameters are shown in Table 1. At the beginning of the protocol, when the animals were divided into their respective groups for subsequent ovariectomy procedure and diabetes induction, body weight was similar between groups. However, at the end of the protocol, the diabetic animals (D, DO and TDO groups) presented reduced body weight when compared to the euglycemic sham operated control animals (E group). Regarding the values of fasting glucose levels measured at the end of the protocol, the diabetic animals (D, DO and TDO groups) had, as expected, higher blood glucose levels than euglycemic sham operated sedentary animals (E group). The DO group presented increased glycemia when compared to both D and TDO groups at the end of the protocol.

**Table 1:** Body weight and glycemia in studied groups.

	E	D	DO	TDO	p
**Body weight (g)**					
**Initial**	218.2±4.1	213.8±1.7	218.4±4.4	222.2±1.8	<0.0001
**Final**	240.6±7.8‡	210.8±9.7*	213.0±5.6*	225.6±5.9*	
**Glycemia (mg/dL)**					
**Initial**	89.0±1.7	403.4±23.0*	408.0±23.9*	410.0±15.2*	<0.0001
**Final**	102.0±4.6	442.7±15.0*	475.0±15.0*#‡	438.0±23.4*†	

Data are reported as mean ± SEM.

* p<0.05 vs. E

# p<0.05 vs. D

†p<0.05 vs. DO

‡ p<0.05 vs. Initial in the same group.

### Exercise capacity

The ovariectomized diabetic group that underwent to combined exercise training showed an increase in running time in the maximal test on treadmill (~66%) and increased maximum load capacity (in the ladder climb, ~63%) when compared to other diabetic animals (D and DO groups) at the end of protocol (Table 2).

**Table 2:** Maximal exercise capacity in the treadmill test (running time) and in the ladder test (load) in studied groups.

	E	D	DO	TDO	p
Running time (min)	11.08±0.86	9.02±0.69*	8.60±0.67*	15.08±0.29*#†	<0.0001
Load (% of body weight)	203±11	174±10	168±11	284±10*#†	<0.0001

Data are reported as mean ± SEM.

* p<0.05 vs. E

# p<0.05 vs. D

†p<0.05 vs. DO.

### Cardiac morphometry and function

In relation to morphometric parameters of the ventricle, the left ventricular mass (LVM) and relative wall thickness (RWT) of the LV were lower in the diabetic animals (D and DO group) when compared to control animals (E group), but these effects were reversed by exercise training (TDO group). The LVM normalized by the body weight was reduced in DO group in relation to DO group, and it was increased in TDO group as compared to all sedentary groups. In addition, an increase in the LV cavity in diastole (LVDIA) was observed in D and DO groups as compared to E and TDO groups. The LVDIA normalized by the body weight was increased in the DO group in relation to the other groups (Table 3).

**Table 3:** Echocardiographic parameters in studied groups.

	E	D	DO	TDO	p
**LVM (g)**	1.09±0.017	0.96±0.006*	0.92±0.019*	1.09±0.024#†	<0.0001
**LVM/body weight**	4.61±0.10	4.44±0.08	4.35±0.04*	5.29±0.04*#†	<0.0001
**LVDIA (cm)**	0.65±0.01	0.72±0.007*	0.71±0.01*	0.63±0.01#†	<0.0001
**LVDIA/body weight**	2.87±0.12	3.05±0.19	3.52±0.14*	3.34±0.07	0.01
**RWT**	0.45±0.01	0.37±0.02*	0.38±0.01*	0.47±0.02 #†	0.0001
**FS (%)**	0.45±0.01	0.41±0.02	0.38±0.02*	0.44±0.01†	0.01
**VCF (circ/sec)**	0.0054±0.0001	0.0048±0.0001*	0.0036±0.0002*#	0.0042±0.0003*#†	0.01
**IVRT (ms)**	23.50±0.69	30.83±1.04 *	31.33±1.40*	26.5±2.09	0.04
**E/A**	1.68±0.14	1.76±0.07	1.66±0.07	1.64±0.10	0.20

Data are reported as mean ± SEM. LVM: left ventricular mass; LVDIA: left ventricular cavity in diastole; FS: fractional shortening; VCF: velocity of circumferential fiber shortening; RWT: relative wall thickness; IVRT: isovolumetric relaxation time; E/A: peak E/peak A relation.

* p<0.05 vs. E

# p<0.05 vs. D

†p<0.05 vs. DO.

Regarding systolic function, the fractional shortening (FS) was reduced in association of diabetes and ovarian hormone deprivation (DO group) in relation to E and DO groups, but this change was reversed in animals submitted to combined exercise training (TDO vs. DO group). There was a reduction in VCF in the diabetic animals (D, DO and TDO group) in relation to the E group. Ovariectomy promoted additional impairment in VCF (DO vs. D group). However, the combined exercise training attenuated this loss in TDO group (vs. DO group) (Table 3).

Diastolic function was impaired in diabetic animals, as demonstrated by increased IVRT in both D and DO groups, when compared to the E group. This change was not observed in the TDO group. The E/A relation was not different between groups (Table 3).

**Fig 1:**
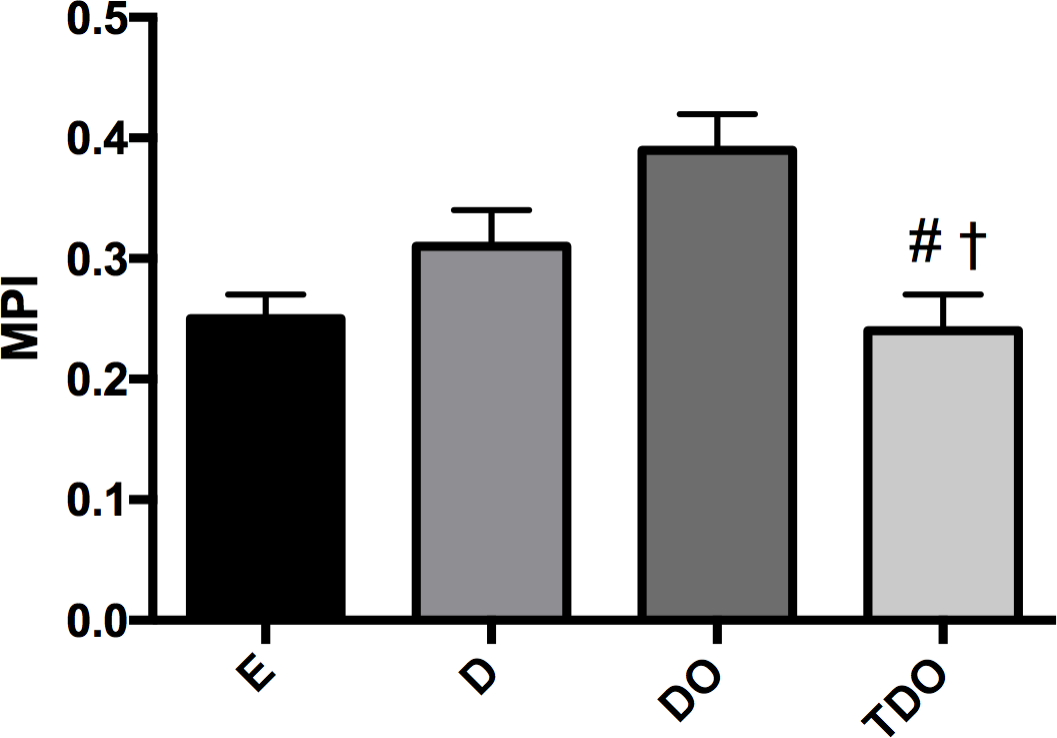
Myocardial performance index (MPI) in studied groups. * p<0.05 vs. E; # p<0.05 vs. D; †p<0.05 vs. DO.

Regarding global function, the MPI, an index that represents the heart's effort to meet the demands of tissue perfusion, showed an increase in D and DO groups when compared to E group. An additional impairment in MPI was observed in DO group (vs. D group). This parameter was normalized by combined exercise training (Fig 1).

### Cardiac inflammation

**Fig 2:**
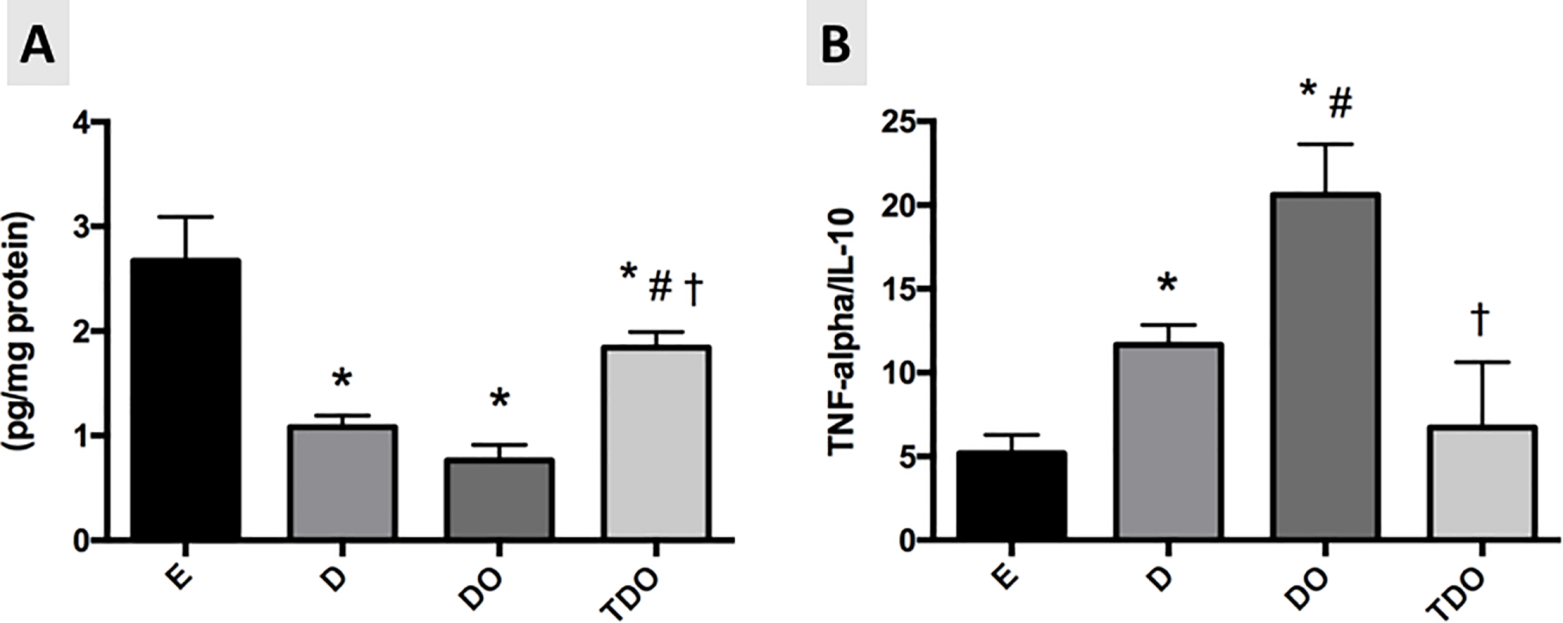
(A) IL-10 levels and (B) TNF-α / IL-10 ratio in cardiac tissue in studied groups. * p<0.05 vs. E; # p<0.05 vs. D; †p<0.05 vs. DO.

The TNF-α levels were not different among studied groups (E: 9.4±4.4; D: 13.2±5.1; DO: 14.7±2.4 and TDO: 12.1±2.3 pg/mg protein). However, the IL-10 levels were decreased in diabetic rats (D: 1.08±0.28; DO: 0.76±0.37 and TDO: 1.84±0.36 pg/mg protein, p<0.05) when compared to E group (2.67±1.05 pg/mg protein). The levels of IL-10 was increased in TDO groups when compared to D and DO groups. Additionally, the TNF-α / IL-10 ratio demonstrated an increase in inflammatory status in diabetic animals (D and DO groups) in relation to control animals (E group). The ovariectomy (DO vs. D group) induced additional impairment in this ratio. The TDO group presented reduced TNF-α/IL-10 ratio as compared to DO group and similar values when compared to E and D groups (E: 5.18±1.34; D: 11.65±3.34; DO: 20.62±3.01 and TDO: 6.72±1.38) (Fig 2).

### Cardiac oxidative stress

Table 4 shows the oxidative stress parameters. Protein oxidation (CARB) was increased in the DO group when compared to D and E groups. The TDO group presented reduced protein oxidation when compared to DO group.

**Table 4:** Oxidative stress parameters in studied groups.

	E	D	DO	TDO	p
**CARB** (nmol/mg protein)	3.00±0.23	5.94±1.59	13.31±3.27*#	9.63±1.48	0.005
**CAT** (nmol/mg protein)	0.80±0.04	0.99±0.01*	1.52±0.13*	1.34±0.20*	0.0009
**SOD** (USOD/mg protein)	16.0±0.6	34.1±4.6*	31.5±2.3*	36.2±5.4*	0.003
**GPx** (nmol/min/mg protein)	32.0±3.6	53.4±6.5	55.9±8.7	57.1±8.6	0.06
**GSH** (mmol/g tissue)	0.258±0.03	0.189±0.02	0.221±0.04	0.271±0.07	0.50
**GSSG** (mmol/g tissue)	0.024±0.005	0.027±0.008	0.038±0.005	0.032±0.006	0.55
**GSH/GSSG**	10.4±1.6	5.8±1.2*	5.8±0.7*	8.6±0.9	0.02

Data are reported as mean ± SEM. *CARB*: *protein oxidation; CAT*: *catalase; GPx*: *glutathione peroxidase; SOD*: *superoxide dismutase; GSH*: *reduced glutathione; GSSG*: *oxidized glutathione*.

* p<0.05 vs. E

# p<0.05 vs. D.

Regarding antioxidant enzymes, the CAT and SOD activities were increased in all diabetic animals (D, DO and TDO groups) when compared to control animals (E group). GPx, reduced glutathione (GSH) and oxidized glutathione (GSSG) were not statistically different among groups. However, the GSH/GSSG was reduced in diabetic animals (D and DO groups) when compared to E group, thus pointing to an impairment in redox balance. The combined exercise training attenuated this reduction in GSH/GSSG in diabetic rats (TDO vs. D and DO groups) (Table 4).

## Discussion

In the present study, we tested the hypothesis that aerobic associated to resistance exercise training, in alternate days, can promote cardiac benefits associated with reduction in inflammation and oxidative stress in cardiac tissue in STZ-induced diabetic ovariectomized rats. The most important finding of this study was the normalization of myocardial performance index in trained rats (TDO group), which represents the cardiac global function, along with an improvement in inflammation and oxidative stress when compared to sedentary diabetic rats (D and DO groups). Furthermore, our study demonstrated that moderate intensity dynamic resistance exercises associated with dynamic aerobic exercise (combined training) was not detrimental to this experimental model of risk factors, which combines diabetes and ovarian hormone deprivation.

The experimental model induced by ovariectomy mimics the status of menopause by suppressing sex hormone levels. This model induces a reduction of circulating levels of ovarian hormones, and metabolic and cardiovascular disorders like those observed in postmenopausal women [17,18,32,34]. In this study, euglycemic rats (E group) showed an increase in body weight during the 8-week follow-up, as previously demonstrated by our group [19,20].

As expected, diabetic animals showed hyperglycemia, according to previously published research by our group [20,21,35–38]. Moreover, the diabetic ovariectomized rats (DO group) presented higher glycemia than the group with isolated diabetes (D group), suggesting that ovarian hormone deprivation influences glucose metabolism in diabetic rats [21]. In fact, there is a very close relationship between estrogen and glucose metabolism. One of the possible mechanisms is that estrogen may determine a low incidence of diabetes through the endothelial function improvement [39,40]. In the absence of estrogen, endothelial dysfunction leads to a change in permeability, reducing peripheral blood flow and, consequently, impairing the action of insulin. Considering that streptozotocin causes partial destruction of pancreatic β-cells and, therefore, animals can survive even without the need for exogenous insulin, it is plausible to hypothesize that a reduction in permeability may be compromising the action of the small amount of insulin produced by the remaining β-cells. Other mechanisms that may be involved in increased glycemia in the DO vs. D group are the evidence that hormone replacement with estrogen can improve insulin sensitivity [41] and also increase hepatic glucose production [41,42], reinforcing the relationship between female sex hormones and insulin response as well as their production.

Regarding exercise performance, in the present study, sedentary diabetic rats (D and DO groups) showed a reduction in running capacity when compared to euglycemic group, which is consistent with previous literature [21]. In addition, reduced VO_2_max, which may be related to impaired ventricular function after diabetes induction, it was also observed in STZ-diabetes experimental model [38,43]. Furthermore, the exercise training protocol applied in our study consisted in a combination of two types of exercise: aerobic (performed on a treadmill) and resistance (performed on a ladder). Studies have suggested that the combination of these types of exercise (combined training) may positively alter the training-induced physiological adaptation. Thus, this type of training has been recommended as an important non-pharmacological tool in the management, prevention and/or attenuation of various risk factors for cardiovascular and metabolic diseases (41–43). In fact, the TDO rats did not show any complication during the combined exercise training period. Moreover, the TDO group demonstrated improvement in physical capacity, as shown by the increase in the running capacity in the maximal treadmill test and in the maximal strength in the maximum load test when compared to other studied groups (E, D and DO groups).

Diabetes mellitus is accompanied by a number of complications due to the abnormal control of glycometabolism and lipid metabolism. Diabetic cardiomyopathy is a condition observed in diabetic individuals, and it is characterized by changes in the myocardial structure and function, regardless of any coronary artery disease and systemic hypertension [44,45]. Concerning cardiac morphometric parameters, diabetic rats (D and DO group) showed reduced relative wall thickness of the LV (RWT) and LV mass (LVM), and increased LVDIA (vs. E group), with an increase in LVDIA normalized by body weight just in DO group in relation to E group, suggesting additional cardiac morphometric impairment after ovarian hormones deprivation. In fact, with regard to the assessment of cardiac morphology, we have previously observed in our laboratory that the experimental STZ induced diabetes promotes a reduction of the wall thickness of the male rat ventricles, thus lending strength to the findings of the present study, which shows decreased RWT and LVM in DO group when compared to control rats. Moreover, other studies have also observed a reduction in heart weight and LVM after 21 days of STZ-induced diabetes in rats [35,36,46,47]. However, combined exercise training was able to avoid such morphometric changes. The trained rats (TDO group) showed reduction in LVDIA and normalization of both LVM and RWT. These findings corroborate previous data from our group, in studies involving aerobic training in an experimental model of myocardial infarction in rats [48] and in STZ-diabetic ovariectomized rats [19].

Concerning systolic function, STZ-diabetic ovariectomized rats showed reduction in FS and VCF when compared to euglycemic rats (E group). In fact, previous data from our group have shown reduced cardiac output in STZ induced diabetic rats (15-day), which may be related to the worsening of systolic function, and probably related to reduced heart rate and myocardial contractility observed in STZ-diabetes [35,36,46,47]. Diastolic dysfunction, characterized by increased IVRT, was observed in all sedentary diabetic animals (D and DO groups) when compared to euglycemic rats (E group). The cardiac dysfunction induced by STZ-diabetes, as demonstrated by the impairment in left ventricular systolic and diastolic functions, also corroborates previously published data [37,46]. In evaluating male STZ-diabetic rats, we have also previously observed systolic and diastolic dysfunctions, both by echocardiography and by direct function measured by LV catheter, similar to those observed in the present study [38].

The combined exercise training protocol prevented some of the deleterious effects observed in sedentary STZ-diabetic ovariectomized rats (DO group). Trained rats showed an increase in VCF and normalization of FS and IVRT. Taken together, these results show improved systolic and diastolic functions after combined exercise training. Interestingly, in a recent study of our group, aerobic exercise training on a treadmill was able to improve systolic and diastolic function in the same model, which was not observed in the trained animals undergoing resistance training protocol alone [19].

In assessing global cardiac function by MPI, a cardiac dysfunction in the diabetic groups (D and DO groups) was found, with additional worsening in the STZ-diabetic ovariectomized rats (DO group). The most exciting aspect of our findings was that trained rats (TDO group) showed an improvement in this parameter, normalizing MPI in relation to euglycemic animals (E group). Note that the MPI is a myocardial stress index and, to our knowledge, has never been evaluated in this model.

Thus, echocardiographic assessments indicate the initial presence of diabetic cardiomyopathy in ovariectomized animals. However, combined exercise training attenuated the impairment in morphological and functional cardiac parameters in ovariectomized diabetic rats. It should be note that a study published by our group in the same model of diabetes and menopause demonstrated that aerobic exercise training (1h/day, 5 days/wk, 8wks) induces attenuation of LV morphometric, systolic and diastolic dysfunctions and oxidative stress; however, resistance exercise training (40 min/day, 5 days/wk, 8wks) did not induce such functional cardiac benefits, despite improving strength and cardiac morphometry and oxidative stress [19]. Although we may expect a more pronounced benefits in cardiac morphometry/function after combined training than isolated aerobic or resistance training, in the present study the total volume of each type of training was ~50% (considering alternate days of aerobic and resistance training) of the applied when we previously studied the effects of just aerobic or resistance training (19). Furthermore, morphometry, systolic and diastolic function were almost normalized after aerobic exercise training in diabetic ovariectomized rats (19). Thus, these aspects, at least in part, may be associate with no markedly exacerbation of combined training effects as compared to aerobic training in this model. However, the combined trained animals (TDO group) showed the sum of beneficial effects in exercise capacity (aerobic + strength improvements) and in cardiac morphometry and function of the isolated types of training. It is important to note that combined exercise training normalized IVRT (vs. E group), which was not observed in diabetic ovariectomized rats submitted to aerobic training (19). This additional benefit in diastolic function probably reflected in improvement of global cardiac function (MPI) in TDO group in the present study.

After demonstrating the presence of cardiac dysfunctions in diabetic ovariectomized rats, we studied the inflammatory and oxidative stress markers in ventricular tissue to understand the mechanisms underlying this important hyperglycemia-induced dysfunction. Regarding the inflammatory mediators, the sedentary diabetic rats (D and DO groups) showed no differences in TNF-α in ventricular tissue, but there was a reduction of IL-10 and an increase in TNF-α/IL-10 ratio in the ventricles of these groups (vs. E group). Moreover, the TNF-α/IL-10 ratio was additionally enhanced in DO group as compared to D group, suggesting additional inflammation after ovarian hormones deprivation. Renna et l. [49] have demonstrated damage to the cell membrane together with increased TNF-α in the vessel in an experimental model of chronic consumption of fructose in SHR rats. Other studies showed a reduction in TNF-α and other interleukins related to inflammation in diabetic groups undergoing aerobic training [50,51]. In this sense, although combined exercise training did not change TNF-α, it did promote an increase in IL-10, an important anti-inflammatory interleukin, and also a significant reduction in TNF-α/IL-10 ratio, suggesting an improvement in the inflammatory status. In this regard, a study has demonstrated that IL-10 prevents oxidative stress induced by TNF-α [51].

In fact, diabetic complications are generally considered to be the result of oxidative stress [12]. Although the primary source of oxidative stress in diabetic cardiomyopathy is yet not fully understood, there seems to be a clear link to hyperglycemia [22]. It has been suggested [52] that the production of activated oxygen species (superoxide, hydrogen peroxide, hydroxyl radical, and singlet oxygen) has a major role in the development of STZ-induced diabetes. In addition, diabetic complications are interrelated with the inflammatory response, and have been shown to be accelerated under a hyperglycemic state for the production of acute response factors in fat cells [53].

Regarding injury markers, we observed an increase in protein oxidation (CARB) in STZ-diabetic ovariectomized animals (DO group) when compared to euglycemic rats (E group). Hydroperoxides have toxic effects on cells both directly and through degradation to highly toxic hydroxyl radicals. Peroxyl radicals can remove hydrogen from lipids, producing hydroperoxides that further propagate the free-radical pathway [13]. The combined exercise training reduced the injury markers (CARB) in TDO group (vs. D and DO groups). Furthermore, the protein oxidation was increased in diabetic ovariectomized rats (DO and TDO groups) when compared to the control animals (E group). This increase might be related to the protein increased damage caused by the ovarian hormone deprivation (DO group) that was not totally reversed by exercise training (TDO group). However, it is important to highlight that these findings of reduction in protein oxidation in TDO group were not observed in previously published paper by our group in ovariectomized rats undergoing isolated aerobic or isolated resistance training [18,19].

Concerning antioxidant defense, we evaluated CAT, which is located in peroxisomes, and decomposes hydrogen peroxide to water and oxygen [13]; and SOD, which converts superoxide anion radicals produced in the body to hydrogen peroxide, thereby reducing the likelihood of superoxide anion interacting with nitric oxide to form reactive peroxynitrite [13]. Both CAT and SOD activity were increased in STZ-diabetic ovariectomized groups (DO and TDO groups) when compared to euglycemic animals (E group). SOD was also increased in D rats when compared to E rats. One possible explanation for the increase in enzymatic antioxidant defense in diabetic rats may be that the combination of risk factors (ovariectomy associated with diabetes), with excessive production of hydrogen peroxide, may have induced led to an increase in the concentration of these enzymes. This improvement probably represent an attempt to counterbalance the increase in reactive oxygen species under hyperglycemic condition [19].

It is therefore important to note that the oxidized glutathione (GSSG) was markedly increased in DO group, and this impairment in a non-enzymatic antioxidant defense may be related to increase in tissue damage (CARB) in this group. However, the most important finding of this study with respect to oxidative stress was the mitigation of the cardiac injury in the glutathione redox balance (GSH/GSSG) in trained rats (TDO group) when compared to other diabetic rats (D and DO groups). This reduction may not be regarded as normalization when compared to the E group. In this aspect, it is important emphasized that previous data from our group demonstrated that isolated aerobic or resistance training attenuated the impairment in cardiac GSH/GSSG, but not normalize this balance (19). Thus, the present findings indicate an additional improvement in a parameter that provides an overview of the balance of oxidative stress after combined exercise training in an experimental model of diabetes and ovariectomy.

It is worth mentioning that the objectives of this study were to evaluate the effects of the combined exercise training specifically on parameters of cardiac morphometry, function, inflammation and oxidative stress in diabetic ovariectomized rats. Thus, our results do not allow to choose the more effective training type (aerobic, resistance or combined) in different populations/conditions, which may actually be dependent on the question being asked.

## Conclusions

These data suggest that the prescription of resistance associated with aerobic training at moderate intensity every other day improve physical capacity, may have a strong positive impact on health and quality of life, does not carry adverse effects and may attenuate diabetic cardiomyopathy, which is more clearly observed after ovarian hormone deprivation. In addition, the attenuation of cardiac morphometric and functional impairments seems to be associated with improvement in markers of inflammation and oxidative stress in the heart in an experimental model of diabetes and menopause. Thus, these experimental results may ground clinical studies involving combined training in the management of cardiovascular risk induced by diabetes and ovarian hormone deprivation.
